# Decentralisation – A Portmanteau Concept That Promises Much but Fails to Deliver? Comment on "Decentralisation of Health Services in Fiji: A Decision Space Analysis"

**DOI:** 10.15171/ijhpm.2016.88

**Published:** 2016-07-02

**Authors:** Stephen Peckham

**Affiliations:** ^1^Centre for Health Services Studies, University of Kent, Canterbury, UK.; ^2^Department of Health Services Research and Policy, London School of Hygiene and Tropical Medicine, London, UK.

**Keywords:** Decentralisation, Decision Space, Organisational Interdependence, Centralisation, Decision-Making, Autonomy

## Abstract

Decentralisation has been described as an empty concept that lacks clarity. Yet there is an enduring interest in the process of decentralisation within health systems and public services more generally. Many claims about the benefits of decentralisation are not supported by evidence. It may be useful as an organising framework for analysis of health systems but in this context it lacks conceptual clarity and particularly often ignores level context issues given the focus on a principal-agent/vertical centre/local axis or other aspects of limits on autonomy such as standards for professional practice. Both these aspects are relevant in discussing the establishment of "decentralised" health centres in Fiji. In the end decentralisation may be nothing more than a useful descriptive label that can be used in an increasingly wide range of ways but actually have little meaning in practice as an analytical concept.

The experiences of decentralisation in Fiji, described by Mohammed et al,^1^ raise some interesting questions about the value of decentralisation as an analytical concept. In particular the findings from the study show how attempts to shift decision-making to more decentralised health units are limited by providing only partial autonomy from central oversight and constrained by relationships at local levels. The authors argue that this limits the benefits of decentralisation for users and providers. While the focus on the vertical principal-agent parameter is useful in terms of exploring how functions are shifted between governmental or administrative layers I would argue that more attention needs to be paid to both the vertical and horizontal relationships between organisation which more fully explain the degree of decision space or what has termed “room for manoeuvre” within which the six “decentralised” health centres operated in the Suva subdivision in Fiji. The health centres provide front-line primary care services and are linked to more local nursing stations. The primary argument of the paper is that due to the limited nature of functions decentralised the benefits of decentralisation will not be realised but it questionable whether these organisational changes in Fiji could, or would, have delivered such benefits in the first place.

Decentralisation is a complex concept that is utilized in a wide range of disciplinary contexts including political science, geography, management studies and organisational theory.^2-5^ It also incorporates concepts of devolution, deconcentration, and delegation with some commentators including privatisation although the latter does not necessarily reflect the two basic typologies relating to geography (spatial dimension) and level (organisational dimension).^2^ However, decentralisation remains a contested concept lacking clarity of definition - as Gershberg put it, “…*the concept of decentralization is a slippery one; a term – like ‘empowerment’ or ‘sustainability’ empty enough on its own that one can fill it with almost anything*” (405),^6^ while others suggest decentralisation remains probably the most confused topic in organization theory and that many of the claims made for the benefits of decentralisation can also be made for centralisation.^7-10^ Mohammed et al describe a process in Fiji where moves towards decentralisation in the period of reform 1999-2004 were subsequently rolled-back in the period 2005-2008 and then promoted again from 2009. This is not surprising as many countries have experienced waves of decentralisation and periods of centralisation and this shifting pattern is often a feature of both well-developed and less developed health systems in countries with lower, and higher incomes.^6,9^ The analysis of decentralisation in Fiji also demonstrates the conceptual problems associated with researching and evaluating decentralisation. Their analytical frame uses Bossert’s concept of decision space within a principal/agent relationship between centre and periphery.^1^ This highlights two very important questions. The first is about the nature of decentralisation itself and the claims made for benefits – particularly in the context of health centres as discussed in the article. The second is whether it might be more relevant to explore the degree of autonomy to act from both vertical and horizontal provides more clarity in exploring policy and decision-making in health systems? One of the key findings of the research on decentralisation in Fiji was that little real decision-making power was decentralised but the authors also refer to the lack of independence of decision-making autonomy even for the function that was decentralised due to local contextual relationships and the potential for autonomous or independent action by local health centres. The authors conclude that the limited transfer of decision-making power limited the achievement of the benefits of decentralisation. While many claims are made about the effectiveness of decentralisation including:

- staff development with increased job satisfaction and loyalty
- improvement in the quality of public services^2^
- increasing experimentation and innovation^11^
- increasing technical efficiency through learning from diversity^9^
- enhancing civic participation neutralising entrenched local elites and thus, increases political stability^3,5,9,10^
- improving governance and public service delivery by increasing the allocative efficiency through better matching of public services to local preferences.^9,12^

However, many claims of the benefits of decentralisation are not supported by strong evidence and may not be specific to decentralisation per se, and in some cases results can be similarly achieved through increased centralisation or through differing combinations of centralisation and decentralisation.^13,14^ For example, Mohammed et al highlight increased participation as a benefit and while the evidence does seem to suggest that there will be increased responsiveness to patients and local communities but responsiveness does not seem to be directly associated with decentralisation.^13^ The health centres are small primary care provider organisations with a small health practitioner staff. Clearly some aspects of healthcare rely on some decentralised activities. For example, the autonomy of patients to participate in health-related decisions does require that the professionals they engage with are able to grant autonomy and respond to patients wishes this does not necessarily rely on the kinds of decentralise functions discussed in their analysis. Thus, patient autonomy is predicated on professional autonomy which itself, may or may not be dependent on decentralised function such as finance, organisation, Human Resources (HR), etc as in the decision space framework (DSF). Irrespective of any organisational or administrative function any discussion of decentralisation needs to include exploring the context of professional practice. Without exploring the nature of professional autonomy and discretion, or examining the impact of professional standards, clinical guidelines, etc, it is not possible to fully explore the degree of choices, autonomy or decision space of the activity (primarily dealing with patients) of the local health centres. Differences in practice between professional groups or in provider organisations is often reliant on professional cultures and practice which can be shaped by organisational contexts but is also dependent on a variety of other factors.^14,15^ There is also an assumption that a more local approach automatically means grater local accountability to the community – and therefore, greater responsiveness. In Fiji, Mohammed et al interestingly note that “…*there is no provision to have the community participate in the health facilities.”* (610).^1^ However, while studies of decentralisation demonstrate a link between increased accountability and responsiveness they do not necessarily demonstrate that these are always associated with each other.^13,14,16^ The crux is how power is shared between powerful interests and patients within the healthcare system.^16^

Mohammed et al utilise Bossert’s ‘DSF,’ an approach that has been widely used to examine decentralisation in developing, low- and middle-income countries.^11,17^ Bossert’s DSF examines the vertical dimensions of decentralisation in terms of the extent to which a range of functions related to finance, service organisation, HR, access and governance are locally or centrally determined.^17^ Here the space for decision-making is determined by the extent to which there is local responsibility and, in cases autonomy, over decisions. In the context of healthcare organisations, Bossert provides a model of autonomy in terms of decentralisation. Using principal-agent theory, this model seeks to explain the interaction between national context and local context in shaping local decision-making which, in turn, shapes the local (organisational) performance. For Bossert the DSF is a means to conceptualise the way in which the processes of decentralisation contribute to its apparent objectives. It does so by distinguishing between three elements:

- *“ the amount of choice that is transferred from central institutions to institutions at the periphery of health systems,*
- *
what choices local officials make with their increased discretion (which may entail innovation, no change or directed change) and
*
- *what effect these choices have on the performance of the health system*” (p. 1513).^17^

Bossert suggests that “*Decentralisation inherently implies the expansion of choice at the local level*” (p. 1518).^17^ The (extent and type of) choices that are permitted by higher authorities (usually central government) through the properties being decentralised and the rules and regulations determine the ‘decision space’ (or rules of the game) that is available locally. Bossert divides the properties being decentralised into functional areas (such as finance or HR) and defines the dimensions of decision space in each of these areas. The functional areas listed are those in which decisions are likely to affect the performance of the health system (loosely defined) in terms of objectives such as equity and efficiency.

Although the DSF recognises the role of local context in determining local choices in decentralised healthcare and reflects the role of performance, it conceptualises local autonomy mainly in the context of vertical decentralisation – the relationship between the centre and the locality. Though this dimension undoubtedly remains crucial, decentralisation also needs to be viewed horizontally.^18-20^ As Fleurke and Willemse state “…*decentralisation or the distribution of responsibilities is organized not only vertically but also horizontally*” (p. 535).^21^ While the vertical central-local axis is of value it provides little assessment of the effectiveness of decentralisation nor conceptualise level context constraints – for example the actions or inactions of other local organisations.

Like many papers exploring decentralisation through application of the DSF, the analysis of decentralisation in Fiji focuses on examining spatial and organisational dimensions assessing where specific functions are located – at a central level/location or dispersed in local areas.^1^ While such an approach provides a useful conceptualisation of decentralisation it does not capture intra- and inter-organisational contexts. The relationship between organisations at any level is important and in healthcare it is clear that local health economies can be a unit of analysis as much as any single organisation. This can perhaps be best understood drawing on Boyne’s concepts of fragmentation and concentration and the relationships between agencies or actors on the vertical and horizontal dimensions.^18^ Boyne argued that the degree of organisational autonomy was not just reliant on the autonomy and responsibility given through vertical structures - ie, given by the principal upper level to the agent lower level – but also by the degree to which autonomy is constrained by other local actors operating at the horizontal level. In this sense decision space is limited by both dimensions with the greater number of “local actors” often limiting the degree of autonomy any single organisation has. This concept has been mainly applied to local governments but can equally be observed within health systems – both in higher and lower income countries. The autonomy of any individual agency depends on the network of relationships at the horizontal level. These include the need to work in partnership with other agencies (for example – working with local hospitals or private partners) or having to operate within existing relationships, an example being local contracts for services with provider agencies. In addition, when examining healthcare organisations it is possible to examine internal decentralisation/centralisation. Thus, a local organisation – in this case the health centres – has its autonomy and capacity to act constrained not just by whether it can make autonomous decisions about finance, resource allocation, access, governance, etc, it may also be constrained by what is possible in their specific local context. In the case of Fiji, these might be related to local context, relationships with nursing stations, local communities, other local services and, given that these are primarily practitioner delivery services, issues of professional competence and guidelines, and relationships with patients. In reality these contextual factors may be more relevant than any degree of decentralised function. Key to the delivery of healthcare to patients will be the degree of autonomy of practitioners in how they treat and care for patients which may be less reliant on having autonomy over finance, organisation, etc.

While autonomy to practice will be key in the context of local health centres, this aspect of decentralisation is not discussed in the analysis of health centres in Fiji as it is focused on organisational factors. The health centres in Fiji were clearly influenced by their local contexts and how they developed their own local partnerships.^1^ In fact part of the decentralisation programme explicitly stated that it appears that local health centres were explicitly empowered to develop their own local partnerships which would significantly shape how autonomous each centre could be, irrespective of any central control or oversight.^1^ This situation has been observed in other countries. Atkinson et al discussed the horizontal dimension of local autonomy in accounting for the “different spaces for autonomy” (p. 626) in Brazil.^22^ They argued that the local context, in terms of “*social organization and political culture*” (p. 626) influenced the actual space available locally and the processes through which this space influences responsiveness, accountability and quality of care (three main expected outcomes of decentralisation). Hospital autonomy reforms faced similar problems in Iran and the United Kingdom.^23,24^ The specific horizontal or local context within which organisations operate means that any analysis of decentralisation needs to take account of additional perspectives including governance and networks, and inter-organisational dependencies. Indeed, the nature of the horizontal level also has relevance in situations of service deconcentration or delegation and even full political devolution. An analysis of these is essential for conceptualising local autonomy and decision space. For example, the governance literature refers the dynamic interaction between and co-existence of collaboration and competition.^23-25^ For example, to achieve their objectives local health centres in Fiji are explicitly supported to develop local partnerships – in many contexts organisations must increasingly collaborate with other agencies, over whom they have no direct or immediate authority. At the same time, as shown in the Fiji experience, their room to manoeuvre, their degree of autonomy, is constrained by the degree of power and responsibility ceded by the centre and the actions of those agencies (in this case for example the hospitals) to provide the decision-making space. In essence the centres have to compete with these other agencies for resources (eg, financial resources from government and HR from the labour market) and also develop partnerships. The inter-organisational literature emphasises the influencing effects of (other) local organisations upon one organisation’s autonomy.^26,27^ These inter-dependencies are highly variable and an organisation’s autonomy and ability to develop services and good care is significantly affected by these dependencies.^26,28-31^

## Conclusion

Decentralisation is not a completely discrete area of research and more attention needs to be paid to how it is utilised as a concept in future practice, policy, and research. As the paper on Fiji very clearly demonstrates, decentralisation is also not a discrete area of activity. The changing nature of the dynamics between different parts of a health system over time – resulting from the combination of multiple centres of direction and regulation (including financial, political, and technical) and multiple strategies emerging among the regulated organisations (including collaboration, compliance, and competition) impact on any system change outcome. In particular, in the context of the discussion of health centres in Fiji, a fuller discussion of the context of professional practice – autonomy, discretion, etc – as well as local relations would have been valuable in gaining a better understanding of the factors that would generate benefits to providers and service users. There is a growing literature on the role that context plays in shaping local decisions.^31-33^ In terms of local autonomy, the decision space afforded vertically by government and horizontally by local organisations shapes both the context within which local agents make decisions and the content/mechanisms which are implemented. In summary, decentralisation viewed in this way reinforces its conception as a process, not simply as a product. The processes of vertical and horizontal decentralisation and other local context dimensions define the “room for manoeuvre” available to local managers and practitioners. Perhaps ultimately we are left with the situation where there is little evidence to suggest that decentralisation is more innovative than centralisation, or vice versa or any other managerial, organisation, policy or professional “innovativation.”^11,13,14^ What remains is the feeling that while decentralisation is presented as a panacea for liberating decision-making and improving health services it is not really more than a portmanteau concept that promises much but, in itself, delivers little.

## Ethical issues

Not applicable.

## Competing interests

Author declares that he has no competing interests.

## Author’s contribution

SP is the single author of the paper.
